# Intranasal dexmedetomidine reduces pain scores in preterm infants during retinopathy of prematurity screening

**DOI:** 10.3389/fped.2024.1441324

**Published:** 2024-08-02

**Authors:** Nurten Ozkan Zarif, Sema Arayici, Kiymet Celik, Zeynep Kihtir, Hakan Ongun

**Affiliations:** Division of Neonatology, Department of Pediatrics, Faculty of Medicine, Akdeniz University, Antalya, Türkiye

**Keywords:** retinopathy of prematurity screening exam, pain management, dexmedetomidine, PIPP score, prematurity

## Abstract

**Background:**

This study aimed to investigate the effectiveness of intranasal dexmedetomidine in reducing pain scores during retinopathy of prematurity (ROP) screening examinations in preterm infants.

**Methods:**

Infants born at ≤32 weeks of gestational age, undergoing routine ROP examinations in the neonatal intensive care unit, were included in the study and divided into two groups: the standard protocol group (*n* = 43) and the dexmedetomidine group (*n* = 56), over a 1-year period. Both groups received standard procedural preparation including swaddling, oral dextrose, and topical anesthesia with proparacaine. The dexmedetomidine group additionally received intranasal dexmedetomidine at a dose of 1 mcg/kg before the procedure. Pain scores (PIPP score), heart rate, respiratory rate, blood pressure, and oxygen saturation were compared at baseline, 1-min, and 5-min during the procedure.

**Results:**

There were no significant differences between the groups regarding descriptive and pre-procedure characteristics. In the dexmedetomidine group, the median (25-75p) PIPP score, heart rate, systolic blood pressure and mean (±SD) respiratory rate measured at the 1st minute of the procedure were significantly lower than those in the standard group [PIPP score 10 (8–13) vs. 14 (10–16), *p* < 0.001; heart rate 165 (153–176) beats/min vs. 182 (17–190) beats/min, *p* < 0.001; respiratory rate 60 (±7) breaths/min vs. 65(±9) breaths/min, *p* = 0.002; systolic blood pressure 78 (70–92) mmHg vs. 87 (78–96) mmHg, *p* = 0.024; respectively] whereas the saturation value was significantly higher (88% (81–95) vs. 84% (70–92), *p* = 0.036; respectively). By the 5th minute of the procedure, the median (25-75p) PIPP score [4 (2–6) vs. 6 (4–10), *p* < 0.001], heart rate [148 (143–166) beats/min vs. 162 (152–180) beats/min, *p* = 0.001] and respiratory rate [56 (54–58) breaths/min vs. 58 (54–62) breaths/min, *p* = 0.034] were significantly lower, and the saturation level was significantly higher [96% (94–97) vs. 93% (91–96), *p* = 0.003] in the dexmedetomidine group. Additionally, the frequency of adverse effects was significantly lower in the dexmedetomidine group compared to the standard protocol group (11% vs. 47%, *p* = 0.001).

**Conclusion:**

Administering intranasal dexmedetomidine before ROP screening examinations was associated with a decrease in pain scores among preterm infants. This suggests its potential as an effective and well-tolerated method for pain management during ROP screenings.

## Introduction

The advancements in neonatal intensive care units (NICUs) and medical treatment methods in recent years have led to notable increases in the survival rates of premature newborns. As a result, particularly very small preemies often require prolonged follow-up in NICUs, where they undergo a multitude of painful interventions. These interventions include vascular punctures, heel punctures for blood sampling, tracheal aspirations, and retinopathy of prematurity (ROP) examinations, among others (1). It is widely recognized that newborns perceive pain and exhibit physiological and hormonal responses to painful stimuli (2, 3). Research indicates that exposure to pain from repetitive procedures can have detrimental effects on cortical thickness, brain volume, and maturation, ultimately leading to impaired motor and cognitive development (1–6). Therefore, effective pain management in newborns is not only clinically necessary but also ethically imperative. Despite the importance of pain management in neonates, the limited number of clinical studies in infants has left the overall effectiveness and safety of analgesic options unclear. This lack of evidence has resulted in a restricted range of available choices for pain management in this vulnerable population. As such, there is a critical need for further research to elucidate the most effective and safe approaches to pain management in newborns undergoing medical procedures (7).

Retinopathy of prematurity is characterized by abnormal vascularization of the developing retina in premature infants, which can lead to vision loss. Early diagnosis and treatment are crucial for mitigating the risk of blindness, especially within the framework of a comprehensive ROP screening program (8). However, the procedures involved in ROP scanning exams, such as the use of mydriatic drugs, insertion of eyelid speculum, forced opening of the eyelids, scleral indentation, and intense illumination, cause acute pain in infants. Moreover, ROP examinations become a recurring source of acute pain for preterm infants, as multiple examinations are often required until retinal vascularization is complete (9–11). Various studies have recommended a range of pain relief approaches during ROP examinations, including both non-pharmacological methods (such as oral sucrose, human milk, non-nutritive sucking, and swaddling) and pharmacological interventions (such as topical anesthesia, paracetamol, and fentanyl). Despite numerous meta-analyses conducted on this topic, there is still no definitive determination regarding the most effective treatment for pain relief during ROP examinations. While there are currently strong recommendations and guidelines for conducting ROP examinations, there is a lack of universally recommended standard treatment protocols for pain relief during these procedures (12–15). For these reasons, further research is needed to establish the most effective and safe pharmacologic agents for pain management during retinopathy of prematurity (ROP) examinations in preterm infants. This research should prioritize identifying agents that not only offer effective pain relief but also minimize the risk of adverse effects, specifically respiratory depression and long-term neurodevelopmental issues (7). Additionally, it's important to explore options that do not require intravenous access, considering the challenges and potential complications associated with this route of administration in preterm infants (16). Investigating alternative pharmacological agents, such as intranasal formulations or other non-invasive delivery methods, could be particularly valuable in this regard (17, 18). These approaches may offer the benefits of effective pain management while reducing the risks associated with invasive procedures like intravenous administration (7, 16).

Dexmedetomidine is a highly selective α-2 agonist known for its sedative, anxiolytic, and analgesic effects (19). Its utilization in NICUs has increased in recent years owing to its ability to provide sedation and analgesia without causing respiratory depression (3). There are several publications in the literature that discuss the intranasal administration of dexmedetomidine, highlighting its efficacy and safety in various clinical settings (20, 21). In this study, our objective was to evaluate the effectiveness of intranasal dexmedetomidine in reducing pain scores during ROP examinations in preterm infants.

## Methods

### Trial design

This study was a single-center, retrospective trial conducted at the level IV NICU of Akdeniz University Hospital, involving preterm infants ≤32 weeks of gestational age. The protocol was approved by the Ethics Committee of Akdeniz University Hospital with the reference number KAEK-17/2023.

### Eligibility criteria

The study included preterm infants with a gestational age of ≤32 weeks who underwent ROP screening. Infants who were mechanically ventilated, hemodynamically unstable, sedated, had a grade >2 intracranial hemorrhage, congenital malformations, genetic syndromes, or neurological dysfunction were excluded from the study.

### Procedure for eye exam

The routine ROP screening protocol adhered the recommendations outlined by the American Academy of Pediatrics, the American Academy of Ophthalmology, and the American Association for Pediatric Ophthalmology and Strabismus guidelines (8).

The ROP screening examination is conducted by the ophthalmologist using the scleral indentation method with a binocular indirect ophthalmoscope after the insertion of a lid speculum. Pupillary dilation is achieved by instilling a combination of phenylephrine (2.5%) and cyclopentolate (1%) drops three times, with intervals of five minutes, beginning one hour before the examination.

According to the standard pain protocol in our unit, during ROP examinations, specific measures are taken to minimize discomfort for the infant. The baby's head is kept in minimal extension, a roll pad is placed under the neck to close the gap between the shoulders and the head, and the arms and legs are swaddled while flexed. Additionally, one minute before the examination, 0.5 ml of oral dextrose (20%) is administered, and just before the procedure, one drop of proparacaine (0.5%) is instilled into the eye for topical anesthesia.

In recent months, our NICU has implemented the administration of intranasal dexmedetomidine as part of our procedural analgesia protocol during ROP screening. The administration procedure for dexmedetomidine involves intranasal delivery using a mucosal atomization device, administered 20 min prior to the eye examination, at a dosage of 1 mcg/kg. This protocol was established based on information gathered from the literature (22, 23). To evaluate the effectiveness of this modification to our protocol, we categorized the infants screened for ROP in our unit over the past year into two groups:

The standard protocol group: This group includes infants who underwent ROP screening using only standard methods without prior procedural analgesia.

The dexmedetomidine group: This group comprises infants who received intranasal dexmedetomidine in addition to the standard methods during ROP screening.

### Pain assessment

The Premature Infant Pain Profile (PIPP) score is a comprehensive system developed to assess pain and/or stress induced by a procedure in infants, based on their behavioral and physiological responses. This scoring system provides a clear and standardized method for quantifying pain levels in infants undergoing procedures, facilitating appropriate interventions for pain management. The PIPP scale includes evaluation of behavioral aspects such as facial movements, physiological parameters like heart rate and oxygen saturation, as well as contextual factors such as gestational age and behavioral state. A total PIPP score ranging from 0 to 6 is considered indicative of mild pain, 7 to 12 signifies moderate pain, and a score of ≥12 indicates severe pain (24).

In our department, the PIPP scale has been extensively utilized for pain assessment due to its simplicity and comprehensiveness, and it is preferred by all healthcare professionals. PIPP scores are evaluated at three key time points: before the procedure, at 1 min after the procedure, and at 5 min after the procedure. This practice represents a standard protocol for assessing pain levels during ROP examinations and evaluating the effectiveness of both non-pharmacological and pharmacological interventions. Evaluations are conducted by the neonatology fellow to ensure adherence to unit protocols. This allows us to effectively monitor and measure pain management outcomes over time.

### Data collection

We documented demographic data of the infants, including birth weight, gestational age, postmenstrual age at the time of the eye examination, and actual weight. Additionally, we recorded pre- and post-procedure vital parameters such as heart rate, respiratory rate, blood pressure, and oxygen saturations (SpO2), along with PIPP scores at 1- and 5-min post-procedure. Furthermore, we documented occurrences of apnea (cessation of breathing lasting more than 20 s, or a cessation of breathing lasting more than 10 s accompanied by bradycardia [heart rate <100 bpm], desaturation [oxygen saturation <80%], or both), desaturation (SPO_2_ < 89%), bradycardia (heart rate <100 bpm), rash (macular or maculopapular skin rash that appeared suddenly and could not be attributed to any other cause), feeding intolerance (vomiting, abdominal distension, or more than 50% residue of the previous feeding), the need for oxygen or respiratory support, or any escalation in the requirement for existing support within 24 h after the procedure.

### Study outcomes

The primary outcome of the study was to evaluate the effectiveness of intranasal dexmedetomidine in managing pain during retinopathy of prematurity (ROP) examinations. The secondary outcome involved assessing any potential adverse effects associated with the use of intranasal dexmedetomidine.

### Statistical analysis

Since there are no reported data regarding the effects of dexmedetomidine on pain during ROP examination as measured by PIPP scores. We considered a three-point reduction in PIPP score during ROP examination between groups to would be clinically significant. To detect this disparity in scores, assuming a standard deviation of 2 and a 10% attrition rate, we estimated a sample size of 43 patients in each group to achieve a study power of 95% at a two-sided significance level of an α error rate of 5%.

The patient data collected for the study underwent analysis using IBM Statistical Package for the Social Sciences (SPSS) for Windows version 17.0 (IBM Corp., Armonk, NY). Descriptive analysis was conducted for the demographic and clinical characteristics of the patients. The normality of group distributions was assessed via the Shapiro-Wilk test. Group comparisons were conducted utilizing either Student's t-test or the Mann-Whitney U test. Descriptive statistics for groups adhering to a normal distribution were displayed as mean ± SD, while those for groups deviating from normal distribution were presented as median (25–75th percentile). For nominal data, group comparisons were carried out using either the chi-square test or Fisher's exact chi-square test. Categorical variables were represented as percentages (%). The significance level (alpha) was set at 0.05.

## Results

A total of 99 examinations meeting the study criteria and with accessible records were evaluated over a 1-year period. Upon analysis, no differences were observed between the groups in terms of demographic characteristics (Table 1). The median gestational age at delivery in the standard protocol group and dexmedetomidine group was 27 (26–28) weeks and 27 (26–29) weeks, respectively (*p* = 0.167). The mean birth weight was 910 (845–1,058) g in the standard protocol group and 998 (855–1,135) g in the dexmedetomidine group (*p* = 0.110). There was no difference between the groups in terms of baseline PIPP scores and vital signs (Table 2). At the first minute after the procedure, the median PIPP score was 14 in the standard protocol group and 10 in the dexmedetomidine group, with a statistically significant difference between the groups (*p* < 0.001). Additionally, compared to the dexmedetomidine group, the standard protocol group exhibited higher median heart rate (182 vs. 165 beats/min, *p* < 0.001), median respiratory rate (65 vs. 60 breaths/min, *p* = 0.005), and median systolic blood pressure (87 vs. 78 mmHg, *p* = 0.024), and exhibited lower median SPO2 (84% vs. 88%, *p* = 0.036). Similarly, at the 5th minute after the procedure, the median PIPP score was 6 in the standard protocol group and 4 in the dexmedetomidine group, with a statistically significant difference between the groups (*p* < 0.001). Although the difference between the groups was significant, the average score of both groups was below the treatment requirement. Additionally, compared to the dexmedetomidine group, the standard protocol group exhibited higher median heart rate (162 vs. 148 beats/min, *p* = 0.001), median respiratory rate (58 vs. 56 breaths/min, *p* = 0.034), and exhibited lower median SPO2 (93% vs. 96%, *p* = 0.003) (Table 2). Furthermore, the frequency of total adverse events in the first 24 h was higher in the standard protocol group compared to the dexmedetomidine group (47% and 11%, respectively, *p* = 0.001). Although apnea was more common in the standard protocol group than the dexmedetomidine group, the difference was not statistically significant (21% vs. 7%, respectively, *p* = 0.07). The frequency of desaturation was higher in the standard protocol group than the dexmedetomidine group (26% vs. 2%, respectively, *p* = 0.001). Bradycardia was observed in only one patient in the dexmedetomidine group (Table 3).

**Table 1:** Demographic data of the study groups.

	Standard protocol group (*n* = 43)	Dexmedetomidine group (*n* = 56)	*P*
	Mean (±SD), Median (25-75p), *N* (%)		
Gestational age at delivery (weeks)	27 (26–28)	27 (26–29)	0.167
Birthweight (grams)	910 (845–1,058)	998 (855–1,135)	0.110
Postmenstrual age (weeks)	34.2 (±2.6)	34.5 (±2.9)	0.577
Gender (male)	24 (56)	35 (62)	0.500
APGAR score (1 min)	5 (4–7)	5 (4–7)	0.865
APGAR score (5 min)	7 (6–8)	7 (6–8)	0.902

**Table 2:** PIPP scores, hearth rate, respiratory rate, oxygen saturation, systolic and diastolic blood pressure values before, 1st and 5th minute after retinopathy examinations.

	Standard protocol group (*n* = 43)	Dexmedetomidine group (*n* = 56)	*P*
	Mean (±SD), Median (25-75p)		
Pre-procedure			
PIPP	4 (3–5)	3 (2–5)	0.087
Hearth rate (beats/minute)	154 (±15)	150 (±15)	0.283
Respiratory rate (breaths/minute)	58 (52–60)	56 (52–58)	0.410
SPO_2_ (%)	95 (93–96)	96 (94–98)	0.268
Systolic blood pressure (mmHg)	75.1 (±11.1)	74.2 (±11.3)	0.685
Diastolic blood pressure (mmHg)	39.8 (±7.6)	38.8 (±7.2)	0.511
1st minute after procedure			
PIPP	14 (10–16)	10 (8–13)	**<0**.**001**
Hearth rate (beats/minute)	182 (170–190)	165 (153–176)	**<0**.**001**
Respiratory rate (breaths/minute)	65 (±9)	60 (±7)	**0**.**002**
SPO_2_ (%)	84 (70–92)	88 (81–95)	**0**.**036**
Systolic blood pressure (mmHg)	87 (78–96)	78 (70–92)	**0**.**024**
Diastolic blood pressure (mmHg)	46.6 (±9.7)	43.8 (±8.8)	0.133
5th minute after procedure			
PIPP	6 (4–10)	4 (2–6)	**<0**.**001**
Hearth rate (beats/minute)	162 (152–180)	148 (143–166)	**0**.**001**
Respiratory rate (breaths/minute)	58 (54–62)	56 (54–58)	**0**.**034**
SPO_2_ (%)	93 (91–96)	96 (94–97)	**0**.**003**
Systolic blood pressure (mmHg)	76.3 (±11.3)	72.2 (±10.6)	0.065
Diastolic blood pressure (mmHg)	40 (34–48)	38 (33–42)	0.219

PIPP, premature infant pain profile; SPO2, oxygen saturation.

Statistically significant results (*P* < 0.05) are indicated in bold.

**Table 3:** Adverse events after retinopathy of prematurity examinations.

	Standard protocol group (*n* = 43)	Dexmedetomidine group (*n* = 56)	*P*
	*N* (%)		
Adverse events, total	20 (47)	6 (11)	**0**.**001**
Apnea	9 (21)	4 (7)	0.07
Desaturation (SPO2<%89)	11 (26)	1 (2)	**0**.**001**
Bradycardia (HR <100/dk)	0	1 (2)	1

Adverse events are the sum of apnea, desaturation, bradycardia; HR, hearth rate; SPO2, oxygen saturation.

Statistically significant results (*P* < 0.05) are indicated in bold.

Adverse events were observed in a total of twenty-six infants, with twenty occurring in the standard protocol group and six in the dexmedetomidine group. Specifically, apnea developed within 24 h following the eye examination in nine infants in the standard protocol group (seven under non-invasive respiratory support and two in spontaneous breathing) and in four infants in the dexmedetomidine group (three under non-invasive respiratory support and one in spontaneous breathing). Fortunately, all cases of apnea were resolved by tactile stimulation, and none of the infants required mask ventilation or intubation. Moreover, four infants in the standard protocol group and one infant in the dexmedetomidine group experienced desaturation while on non-invasive respiratory support, necessitating a 10% increase in FiO2. Additionally, seven infants in the standard protocol group experienced desaturation while in spontaneous breathing, requiring additional oxygen support. Furthermore, in one infant in the dexmedetomidine group, the heart rate decreased to between 95 and 100 beats per minute. However, it resolved spontaneously within 30 s without requiring any intervention. All desaturations and bradycardia occurred within one hour after the eye examination (Table 3).

## Discussion

This study represents the first evaluation of intranasal dexmedetomidine as an analgesic agent during the ROP screening examination. Our findings demonstrate that intranasal dexmedetomidine effectively alleviates examination-related pain in infants without significant side effects. Moreover, our study underscores the assertion that the ROP screening examination is indeed a painful procedure, and pain cannot be adequately controlled with topical anesthetics and non-pharmacological methods alone. In line with prior literature highlighting the sedative effects of dexmedetomidine during procedural procedures (22, 23), our objective was to examine the analgesic effect of intranasal dexmedetomidine in patients within our unit. The outcomes of our study revealed lower pain scores with the administration of dexmedetomidine, and importantly, no significant side effects were observed.

The retinopathy of prematurity (ROP) examination is acknowledged as a painful procedure that could potentially contribute to long-term neurodevelopmental issues in preterm infants (5, 8). In existing literature, there is no universally recognized single method for managing pain during the ROP examination. Instead, a combination of non-pharmacological and pharmacological agents is typically recommended (9–11). Studies have demonstrated that while non-pharmacological agents can reduce pain to some extent, they do not completely eliminate it. Although these methods may assist in distracting infants and blocking pain transmission to the cerebral cortex, they fall short of providing comprehensive pain relief (9). A meta-analysis conducted by Disher et al. (11) underscored the persistent challenge of effectively managing pain during ROP examinations. Despite the implementation of multisensory interventions, the analysis found that the pain scores of most infants undergoing ROP examinations remained elevated, often exceeding a threshold of twelve. This highlights the urgent need for the development of additional methods, in conjunction with non-pharmacological approaches, to ensure effective pain management during ROP screening.

Benzodiazepines and opioids, commonly used pharmacological agents for analgesia, have limited applicability in the context of retinopathy of prematurity (ROP) examinations due to their potential to induce respiratory depression and the typically required intravenous administration. In addition to short-term negative effects such as apnea, hypotension, and urinary retention, long-term neurodevelopmental side effects including intraventricular hemorrhage, periventricular leukomalacia, neuroapoptosis, paradoxical agitation, and behavioral and cognitive disorders have also been reported with these agents (3, 15, 25). Hartley et al. (7) conducted a study comparing oral morphine with a placebo for pain control during ROP examinations in preterm infants. They found no difference in pain scores between the groups; however, the study was halted due to the emergence of new-onset apnea or an increase in the number of apnea episodes in infants within the morphine group. Sindhur et al. (26) compared fentanyl which had better respiratory safety than morphine with placebo, in infants during ROP examinations. Despite not completely eliminating pain, the study reported a significant reduction in pain scores with fentanyl. Kara et al. (27) compared sucrose, intravenous fentanyl, and intranasal fentanyl during ROP examinations but found that the three methods were not superior to each other.

It is essential for the analgesic agent to deliver effective pain relief without inducing respiratory depression, exhibiting neurotoxicity, or necessitating intravenous access for preterm infants (7, 16–18). Recently, dexmedetomidine has emerged as a promising agent for procedural sedation and analgesia, particularly due to its ability to provide these effects without causing respiratory depression. This characteristic has made it a preferred choice in intensive care units in recent years (17, 28). Dexmedetomidine exerts its analgesic and sedative effects by stimulating α2 receptors in the locus coeruleus of the brainstem. Moreover, studies have suggested that dexmedetomidine may possess neuroprotective properties, attributed to its anti-inflammatory and anti-apoptotic effects (17, 28–30). While the use of dexmedetomidine in pediatric and adult patients is well established, there is relatively limited data available regarding its application in the neonatal population. Despite not being approved by the Food and Drug Administration (FDA) for use in neonates, the off-label use of dexmedetomidine has been increasing in recent years (31–33). This highlights the need for further research to better understand its safety and efficacy profile in neonates, particularly in the context of procedures such as ROP examinations. Such investigations could provide valuable insights into the potential role of dexmedetomidine in improving pain management and outcomes in this vulnerable population.

In a study comparing the use of dexmedetomidine and fentanyl for sedation in extremely preterm infants, Nakauchi et al. (34) reported that the dexmedetomidine group required less additional sedation. Furthermore, there was no significant difference between the two groups in terms of death and a developmental quotient below 70 at a corrected age of 3 years. Leister et al. (35) conducted a retrospective evaluation of preterm and term infants who underwent sedation with dexmedetomidine for MRI. They suggested the safety of dexmedetomidine for procedural sedation in both preterm and term infants. Portelli et al. (3) published a review assessing the utilization of dexmedetomidine in mechanically ventilated newborns. The review highlighted that, neonates in the dexmedetomidine group experienced a shorter time to extubation, lower pain, and higher sedation scores. Furthermore, O'Mara et al. (36) reported a favorable safety profile of dexmedetomidine, with no significant adverse events necessitating abrupt discontinuation.

Intranasal drug administration provides an alternative to the intravenous route, offering a noninvasive approach that eliminates the need for intravenous access. This method is considered safe and effective, as it bypasses hepatic first-pass metabolism, theoretically resulting in plasma levels equivalent to those achieved with intravenous dosing (17, 18). The intranasal route is commonly used for administering dexmedetomidine in children, with numerous studies exploring its efficacy for procedural sedation and premedication in pediatric patients. A recent meta-analysis indicated that intranasal dexmedetomidine achieves successful sedation in premedication, surpassing other intranasal or oral agents (20, 21). In their review, Lewis et al. (22) asserted that intranasal administration of dexmedetomidine for sedation in children is a simple and effective method, providing reliable sedation. Another systematic review and meta-analysis concluded that intranasal dexmedetomidine is more effective than oral chloral hydrate and oral midazolam for procedural sedation in children (23, 37). In recent times, the use of intranasal dexmedetomidine in infants has garnered attention. While there is no prospective study in the literature on the use of intranasal dexmedetomidine for procedural anesthesia in newborns, retrospective evaluations and a few case series presentations are available. Bua et al. (38) presents their experience with the use of intranasal dexmedetomidine for sedation in preterm neonates undergoing brain MRI at term equivalent age, suggesting that intranasal dexmedetomidine could serve as an alternative for procedural sedation in preterm infants. Zhou et al. (39) employed intranasal dexmedetomidine for the sedation of newborn infants undergoing MRI in their unit and reported successful sedation without bradycardia or other side effects.

The primary concerns associated with dexmedetomidine use include bradycardia and hypotension. In our study, we observed a transient episode of bradycardia with a heart rate between 95 and 100 beats per minute for thirty seconds in an infant from the dexmedetomidine group. However, this bradycardia resolved spontaneously without intervention. Additionally, hypotension was not observed in any of the infants receiving dexmedetomidine in our study. However, although studies have shown that it is neuroprotective by reducing apoptosis, this has not yet been proven in newborns. In their study on rats, Pancara et al. (40) reported that dexmedetomidine increased apoptosis when used in long-term infusions and high doses. Cordes-Ledesma et al. (41) reported that dexmedetomidine caused a decrease in activity in amplitude-integrated electroencephalography (aEEG) in preterm infants. Therefore, although dexmedetomidine seems to be a good option for neonatal anesthesia and sedation due to its low side effect profile, its potential negative consequences remain unclear. Well-designed, randomized controlled clinical studies on the use of dexmedetomidine in newborns are needed.

One of the most significant limitations of our study was its retrospective nature. Additionally, it could not be blinded, and PIPP scores were evaluated by a single expert. Another limitation was that ROP examinations were not consistently performed by the same ophthalmologist throughout the study period. Furthermore, clinical experience with intranasal administration of dexmedetomidine in the neonatal population is limited, and its pharmacokinetics in neonates are not well understood. Consequently, the appropriate dose and timing of administration have been derived from a limited number of studies. These limitations highlight the need for further prospective research to better understand the efficacy, safety, and optimal dosing of intranasal dexmedetomidine in the neonatal population undergoing painful procedures such as ROP screening.

## Conclusion

At present, a definitive consensus on the pain relief protocol for preterm infants during the ROP examination, aside from topical anesthesia and nonpharmacological methods, is lacking. Intranasal dexmedetomidine emerges as a promising alternative to opioids or benzodiazepines for effective pain management without inducing apnea and without the need for intravenous access. This study concludes that intranasal dexmedetomidine may offer beneficial short-term effects without significant adverse events when utilized for procedural pain management in the NICU during ROP screening exams. However, routine recommendations for clinical practice await adequately powered and well-designed randomized controlled studies that include measures of long-term efficacy, safety, and neurodevelopmental evaluation before dexmedetomidine can be considered for routine use in ROP screening examinations. Further research is essential to establish the full potential and safety profile of intranasal dexmedetomidine in this context.

## Data Availability

The raw data supporting the conclusions of this article will be made available by the authors, without undue reservation.
